# Thyroid Hormone Protects from Fasting-Induced Skeletal Muscle Atrophy by Promoting Metabolic Adaptation

**DOI:** 10.3390/ijms20225754

**Published:** 2019-11-15

**Authors:** Sarassunta Ucci, Alessandra Renzini, Valentina Russi, Claudia Mangialardo, Ilenia Cammarata, Giorgia Cavioli, Maria Giulia Santaguida, Camilla Virili, Marco Centanni, Sergio Adamo, Viviana Moresi, Cecilia Verga-Falzacappa

**Affiliations:** 1 Pasteur Institute, 00161 Rome, Italy; ucci.sara@gmail.com (S.U.); vale-russi@hotmail.it (V.R.); claudiamangialardo@tiscali.it (C.M.); ileniacammarata89@gmail.com (I.C.); cecilia.vergafalzacappa@uniroma1.it (C.V.-F.); 2 DAHFMO Unit of Histology and Medical Embryology, Interuniversity Institute of Myology, Sapienza University of Rome, 00161 Rome, Italy; alessandra.renzini@uniroma1.it (A.R.); cavioli.1703739@studenti.uniroma1.it (G.C.); sergio.adamo@uniroma1.it (S.A.); 3 Department of Medico-Surgical Sciences and Biotechnologies Sapienza University of Rome, 04100 Latina, Italy; mariagiulia.santaguida@uniroma1.it (M.G.S.); camilla.virili@uniroma1.it (C.V.); marco.centanni@uniroma1.it (M.C.)

**Keywords:** muscle atrophy, metabolic reprogramming, thyroid hormone

## Abstract

Thyroid hormones regulate a wide range of cellular responses, via non-genomic and genomic actions, depending on cell-specific thyroid hormone transporters, co-repressors, or co-activators. Skeletal muscle has been identified as a direct target of thyroid hormone T3, where it regulates stem cell proliferation and differentiation, as well as myofiber metabolism. However, the effects of T3 in muscle-wasting conditions have not been yet addressed. Being T3 primarily responsible for the regulation of metabolism, we challenged mice with fasting and found that T3 counteracted starvation-induced muscle atrophy. Interestingly, T3 did not prevent the activation of the main catabolic pathways, i.e., the ubiquitin-proteasome or the autophagy-lysosomal systems, nor did it stimulate de novo muscle synthesis in starved muscles. Transcriptome analyses revealed that T3 mainly affected the metabolic processes in starved muscle. Further analyses of myofiber metabolism revealed that T3 prevented the starvation-mediated metabolic shift, thus preserving skeletal muscle mass. Our study elucidated new T3 functions in regulating skeletal muscle homeostasis and metabolism in pathological conditions, opening to new potential therapeutic approaches for the treatment of skeletal muscle atrophy.

## 1. Introduction

Thyroid hormones (THs), T3 and T4, play critical roles in growth, differentiation, and metabolism. They enter the cell through molecular transporters [1] and bind thyroid receptors (TRs), which classically act as transcription factors [2]. Besides, a “non-genomic action” of THs, involving non-nuclear TRs or different TH-binding proteins, has been reported, which does not target gene transcription, acting both in the cytoplasm and in the nucleus [3,4]. T3-dependent activation of the phosphatidylinositol 3-kinase (PI3K)/AKT pathway has been evidenced in various cell types, leading to cell death prevention in many organs [5,6,7,8]. The T3-mediated Akt activation also ameliorates pancreatic islets function and vitality in both ex vivo and in vivo models [9,10]. Our group recently also highlighted the mitogenic and antiapoptotic T3 action on granulosa cells and tenocytes [8,11,12].

THs target also skeletal muscle tissue, regulating oxygen consumption, fiber composition, calcium mobilization, and glucose uptake [13,14]. Adequate serum TH levels are crucial for skeletal muscle homeostasis since muscle performance is impaired in both hypo- and hyperthyroidism [15].

Skeletal muscle mass and composition are continuously modulated during development or upon different stimuli, including pathological conditions and aging [16,17]. For example, anorexia, a consequence of several chronic pathophysiological processes or aging, leads to muscle atrophy. Skeletal muscle atrophy is caused by a decrease in cell size, with concomitant loss of organelles, cytoplasm, and proteins [18], and by a reduction in myofiber number. Regardless of the specific triggering stimulus, muscle atrophy is characterized by an imbalance between protein synthesis and degradation [19,20]. Muscle atrophy triggered by food deprivation shares common transcriptional reprogramming responses with the wasting induced by other atrophic stimuli, such as the activation of the ubiquitin-proteasome or the autophagy-lysosomal system, as well as the decreased expression of genes involved in transcription and translation [21]. Differently from other atrophic stimuli, in starvation-induced muscle atrophy, protein breakdown is necessary as a source of amino acids for gluconeogenesis. Moreover, altered levels of insulin growth factors and glucocorticoids contribute to muscle atrophy [22], while Nuclear Factor kB is not activated in skeletal muscle upon starvation [23]. Both the ubiquitin-proteasome and the autophagy-lysosomal pathways are activated by the transcription factor FoxO3 [24,25], which is negatively regulated by AKT. 

In the ubiquitin-proteasome system, proteins tagged for degradation are targeted through covalent attachment of multiple ubiquitin molecules. Once the protein is poly-ubiquitinated, it is docked to the proteasome for degradation. The E3 ubiquitin ligases, which catalyze the attachment of the ubiquitin from the E2 ubiquitin ligase to the substrate, are considered the rate-limiting step of the ubiquitination process, which directly reflects the proteasome-dependent protein degradation. The muscle-specific E3 ubiquitin ligases, Atrogin-1 and MAFbx, defined as atrogenes being the common downstream effectors of different atrophic stimuli [26], are up-regulated in catabolic conditions and considered markers of muscle atrophy [27]. 

Autophagy is a well-conserved catabolic process that promotes cellular homeostasis and ensures cell survival, being present at low levels in physiological conditions. In response to starvation or food deprivation, cells increase the formation of membrane-bound autophagosomes to engulf cytoplasmic proteins, lipids, and organelles [25,28,29,30]. The cargoes are delivered to lysosomes for degradation, which provides metabolites to sustain energy demands in nutrient-limiting conditions.

Skeletal muscle is composed of different fiber types with distinct contractile and metabolic properties. Based on metabolism, myofibers are classified into oxidative, intermediate, and glycolytic ones [31]. Oxidative fibers are characterized by a higher number of mitochondria and a slower metabolism. The transcription factor Peroxisome proliferative activated receptor Gamma Coactivator 1 alpha (PGC-1α) promotes the expression of several genes involved in mitochondriogenesis and oxidative metabolism, also controlling various signaling pathways involved in skeletal muscle wasting [32,33,34]. Fiber metabolism is subject to changes depending on the environment. For instance, the slow phenotype depends on slow-type innervation and appropriate intracellular calcium signaling [35,36,37]; while THs influence the shift from slow to fast contractile function [35,36,38]. Despite the role of THs in regulating skeletal muscle metabolism and development has been described, nothing is known about their role in regulating muscle homeostasis in a pathological state such as starvation-induced muscle atrophy. 

Here, we show that T3 counteracted starvation-induced skeletal muscle atrophy by modulating type-specific fiber metabolism without affecting the activation of the ubiquitin-proteasome or the autophagic-lysosome systems, or by increasing protein synthesis. Our finding not only describes a previously uncharacterized role of T3 in modulating muscle homeostasis but also reveals a novel mechanism of muscle mass preservation, independent of modulating the catabolic or the anabolic pathways.

## 2. Results

### 2.1. T3 Counteracts Starvation-Induced Skeletal Muscle Wasting

With the aim of defining the role of thyroid hormone in starvation-induced skeletal muscle atrophy, adult mice were randomly divided into four groups: half of them were starved (STV) for 48 h, while the rest were fed ad libitum as controls; in each condition, mice were treated with daily intraperitoneal injections of either T3 [100 μg/kg BW], or vehicle in controls, as previously done [39]. To validate T3 administration, we checked the expression of several known T3-target genes, i.e., GLUT4, UCP3 [13], MyoD [40], Myh7 [41], and Myh1 [42], in Tibialis anterior (TA) skeletal muscle of CTR and T3-treated mice, 24 h after T3 administration, by real-time PCR. As expected, the expression of all these genes was significantly modulated in T3-treated mice (Table 1). Importantly, none of the treatments significantly affected circulating T3 levels in mice, evaluated 24 h after the second and last injection (Figure S1a). Moreover, the expression of type-2 iodothyronine deiodinase (Dio2) resulted significantly reduced by STV; however, the T3 administration did not importantly change its expression (Figure S1b). 

**Figure 1:**
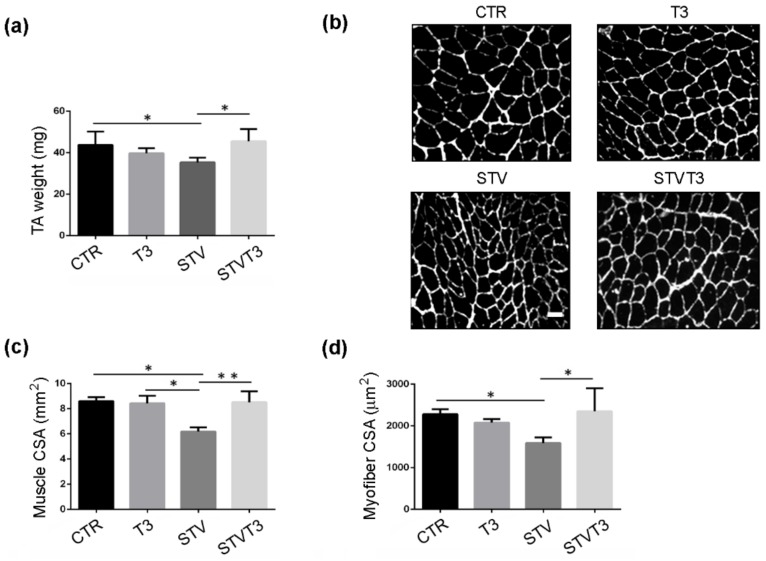
T3 counteracts starvation-induced skeletal muscle loss in Tibialis Anterior. (**a**) TA muscle weight after 48 h of indicated treatments. *n* = 5 mice per each condition. Data are presented as means ± SD. STV and T3 significantly interact (*p* = 0.011) by two-way ANOVA; * *p* < 0.05 by post hoc Tukey’s HSD test. (**b**) Representative pictures of TA muscles with laminin staining, 48 h after starvation. Scale bar = 20 μm. (**c**) Morphometric analyses of the whole TA CSA. *n* = 4 mice per each condition. Data are presented as means ± SD; STV has a significant effect (*p* = 0.038); STV and T3 significantly interact (*p* = 0.029) by two-way ANOVA; * *p* < 0.05; ** *p* < 0.01 by post hoc Tukey’s HSD test. (**d**) Morphometric analyses of myofiber CSA. *n* = 4 mice per each condition. Data are presented as means ± SD; STV and T3 significantly interact (*p* = 0.006) by two-way ANOVA; * *p* < 0.05 by post hoc Tukey’s HSD test.

TA and soleus skeletal muscles were isolated, weighed, and examined by histological and morphometric analyses, 48 h after treatments. The Control (CTR) TA weight resulting was significantly decreased by STV, while T3 treatment prevented STV-dependent TA weight loss, without significantly affecting muscle weight per se (Figure 1a). Muscle cryosections were stained for laminin, to visualize the myofiber size better. Muscle histology revealed that, while STV visibly reduced myofiber size, T3 per se did not affect it but, in combination with starvation, rescued myofiber atrophy (Figure 1b). Morphometric analyses of the whole TA and of single myofiber cross-sectional area (CSA) confirmed that STV induced a significant reduction in the mean of CSA, as expected, and T3 inhibited such decrease, despite the pro-atrophic stimulus (Figure 1c,d).

**Figure 2:**
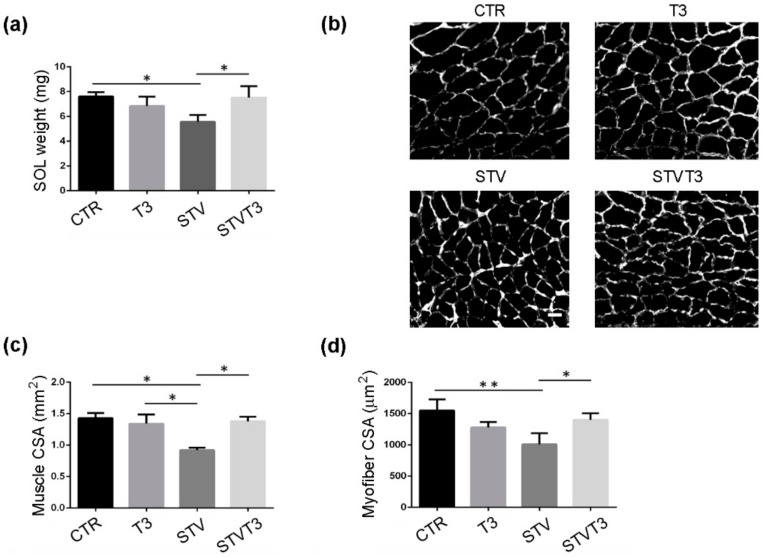
T3 counteracts starvation-induced skeletal muscle loss in soleus. (**a**) Soleus muscle weight after 48 h of indicated treatments. *n* = 3 mice per each condition. Data are presented as means ± SD. STV and T3 significantly interact (*p* = 0.003) by two-way ANOVA; * *p* < 0.05 by post hoc Tukey’s HSD test. (**b**) Representative pictures of soleus muscles with laminin staining, 48 h after starvation. Scale bar = 20 μm. (**c**) Morphometric analyses of the whole soleus CSA. *n* = 3 mice per each condition. Data are presented as means ± SD; STV has a significant effect (*p* = 0.035); STV and T3 significantly interact (*p* = 0.016) by two-way ANOVA; * *p* < 0.05 by post hoc Tukey’s HSD test. (**d**) Morphometric analyses of myofiber CSA. *n* = 3 mice per each condition. Data are presented as means ± SD; STV has a significant effect (*p* = 0.017); STV and T3 significantly interact (*p* = 0.003) by two-way ANOVA; * *p* < 0.05; ** *p* < 0.01 by post hoc Tukey’s HSD test.

Similar results were obtained from soleus muscle: STV significantly reduced muscle weight, while T3 counteracted muscle weight loss without affecting muscle mass per se (Figure 2a). Soleus histological and morphometric analyses confirmed that STV significantly decreased the whole soleus and single myofiber CSA, with respect to CTR, while T3 per se did not affect muscle or myofiber CSA, in combination with starvation, prevented soleus atrophy (Figure 2b–d).

Considering that STV and T3 similarly affected both TA and soleus muscles, we pursued our analyses on TA muscles, searching for the molecular mechanism underlying this striking phenotype.

### 2.2. Thyroid Hormone Does Not Modulate the Catabolic Pathways Induced by Starvation

**Figure 3:**
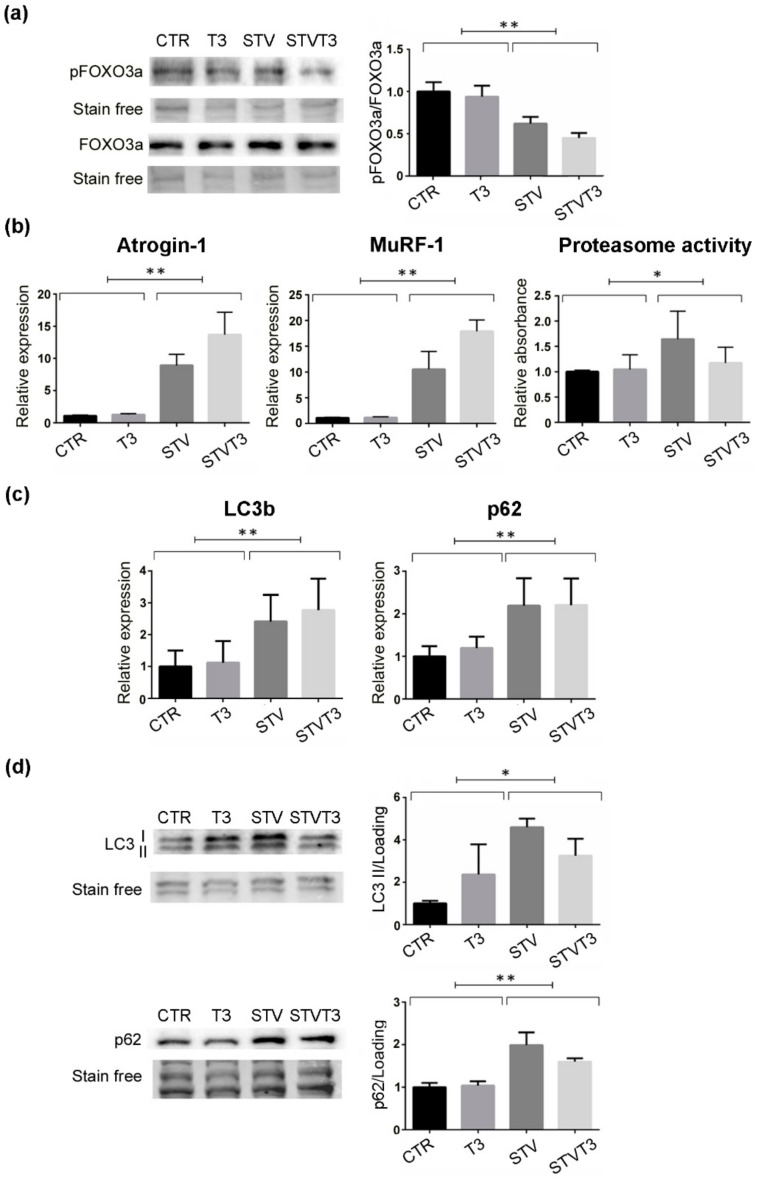
T3 does not prevent catabolic pathway activation in skeletal muscle upon starvation. (**a**) Western blot and densitometric analysis of pFOXO3a/FOXO3a protein levels in TA, 24 h after treatments. Stain-free protein bands were used as a loading control. *n* = 4 mice per each condition. Data are presented as means ± SEM; STV has a significant effect (** *p* = 0.0001) by two-way ANOVA. (**b**) Atrogin-1 and MuRF-1 expression by real-time PCR, 24 h after treatments. *n* = 6 mice per each condition. Data are presented as means ± SD; STV has a significant effect (** *p* < 0.0001) by two-way ANOVA. Proteasome activity assay, 48 h after starvation; *n* = 6 mice per each condition. STV has a significant effect (* *p* = 0.021) by two-way ANOVA. (**c**) LC3b and p62 expression by real-time PCR, 24 h after treatments. *n* = 5 mice per each condition. Data are presented as means ± SD; STV has a significant effect (** *p ≤* 0.001) by two-way ANOVA. (**d**) Representative western blot and densitometric analyses of LC3b I/II and p62 protein levels in TA, 24 h after treatments. Stain-free protein bands were used as a loading control. n = 4 mice per each condition. Data are presented as means ± SEM; STV has a significant effect (* *p* = 0.0149; ** *p* = 0.001) by two-way ANOVA.

Muscle wasting is caused by increased muscle protein breakdown, due to the activation of two major pathways, the ubiquitin-proteasome and the autophagic-lysosomal systems [27]. Since FOXO3 is an upstream regulator of both these catabolic pathways [27], its phosphorylation status was monitored in our experimental system. Strikingly, pFOXO3a/FOXO3a levels were significantly lowered by starvation, regardless of T3 treatment (Figure 3a and Figure S2a,b). Since the ubiquitin-proteasome system is one of the main catabolic pathways responsible for muscle atrophy, we checked its activation in our experimental conditions. We first monitored two muscle-specific E3-ubiquitin ligases, atrogin-1, and MuRF-1, whose expression is importantly up-regulated in catabolic conditions [26]. Coherently, both these genes were significantly induced by STV, as expected, regardless of T3 treatment (Figure 3b). Moreover, we quantified the proteasome activity. While STV induced a significant increase in the proteasome activity, as expected, T3 did not prevent its activation (Figure 3b). Importantly, T3 did not induce statistically significant variations in any of the variables measured. 

The thyroid hormone has been proved to stimulate autophagy in skeletal muscles [43]. To evaluate the effects of T3 administration on autophagy in our experimental system, we measured the expression and protein levels of two main autophagic markers by real-time PCR and western blot analyses. Starvation significantly induced the expression and protein levels of both LC3b and p62, while T3 did not modulate either their expression or their protein amount in skeletal muscle (Figure 3c,d and Figure S2c). These data indicate that T3 rescued starvation-induced skeletal muscle atrophy without altering the activation of the ubiquitin-proteasome or autophagic pathways.

### 2.3. Thyroid Hormone Does Not Induce Skeletal Muscle Synthesis

**Figure 4:**
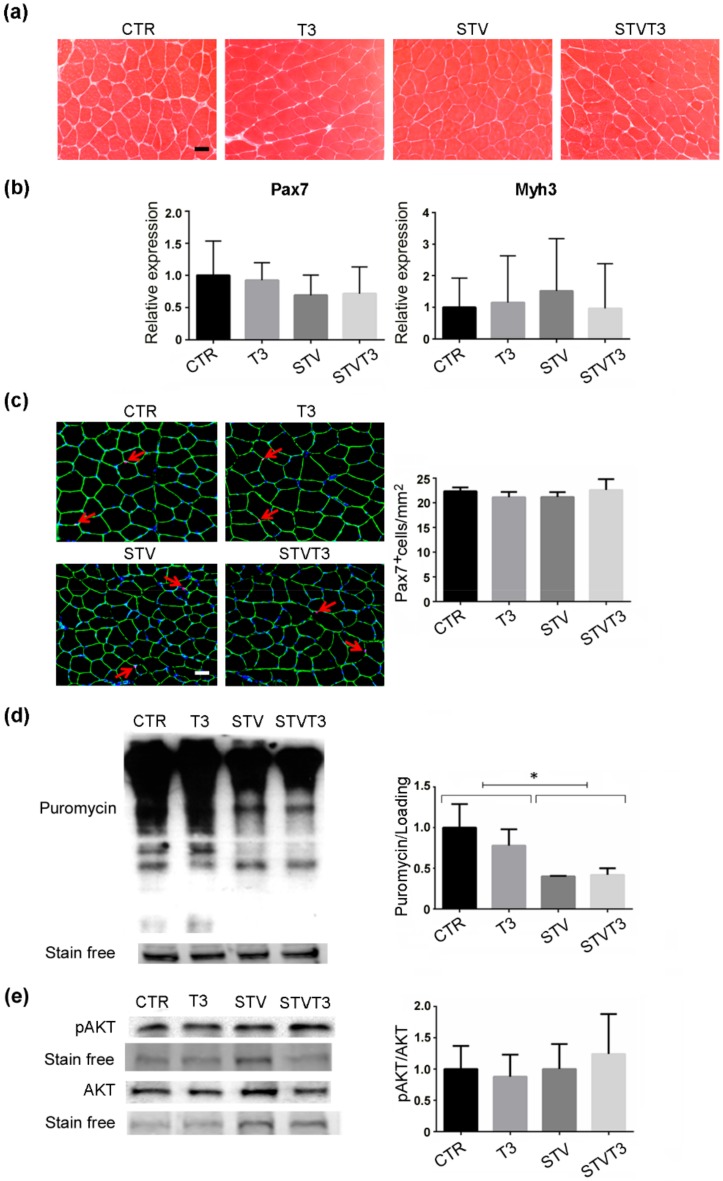
T3 does not increase skeletal muscle anabolism in skeletal muscle following starvation. (**a**) Representative histological sections of TA muscles, stained with hematoxylin and eosin, showing no evident sign of de novo muscle synthesis, 48 h after treatments. Scale bar = 20 μm. (**b**) Pax7 and Myh3 mRNA expression, by real-time PCR, 24 hours after treatment. *n* = 6 mice per each condition. Data are presented as means ± SD. (**c**) Representative images of TA muscle stained for Pax7 (red arrows), laminin (green), and Hoechst (blue). Scale bar = 20 μm. Quantification of the total number of Pax7^+^ cells/mm^2^. *n* = 3 mice per each condition. (**d**) SUnSET assay 48 h after treatments. Stain-free protein bands were used as a loading control. *n* = 4 mice per each condition. Data are presented as means ± SD; STV has a significant effect (* *p* < 0.008) by two-way ANOVA. (**e**) Representative blot and relative densitometric analysis of phospho-Akt and Akt protein levels in TA, 24 h after treatments. Stain-free protein bands were used as the loading control. *n* = 4 mice per each condition. Data are presented as means ± SEM.

Increased muscle regeneration, which mainly depends on satellite cells, counteracts skeletal muscle loss. Since T3 has been reported to potentiate satellite cell differentiation and muscle regeneration upon injury [44,45], we wondered if T3 could counteract STV-mediated muscle atrophy by triggering de novo myogenesis. Muscle synthesis was evaluated in our experimental conditions, without inducing muscle damage, first by histological analyses. No evidence of newly-formed, centronucleated myofibers was observed in any of the conditions analyzed (Figure 4a). Moreover, we analyzed the expression of Pax7, a satellite cell marker, and embryonic myosin heavy chain (Myh3), a marker of de novo myogenesis. As shown in Figure 4b, no significant changes in the expression of these markers were detected among experimental conditions. Pax7 protein expression was further evaluated by immunofluorescence analyses in our experimental conditions. No significant changes in the number of pax7^+^ cells were reported among treatments (Figure 4c).

The protein synthesis rate in skeletal muscles was quantified by the SUnSET technique [46]. Western blot analyses detecting puromycin showed that starvation significantly decreased in vivo protein synthesis in whole muscles, while T3 did not affect the rate of synthesis, regardless of the pro-atrophic stimulus (Figure 4d and Figure S2d). We further analyzed the activation of Akt in our experimental conditions, due to its well-known role in promoting protein synthesis in skeletal muscle [47] and the well-documented action of T3 in activating the Akt signaling in other tissues [5,6,7,8]. Phospho-Akt and Akt protein levels did not significantly differ among our experimental conditions by Western blot analyses (Figure 4e and Figure S2a,b). These results demonstrate that there is no involvement of de novo muscle synthesis in T3 treated muscles that may account for the rescue of starvation-induced skeletal muscle atrophy.

### 2.4. T3 Modulates Skeletal Muscle Gene Expression

**Figure 5:**
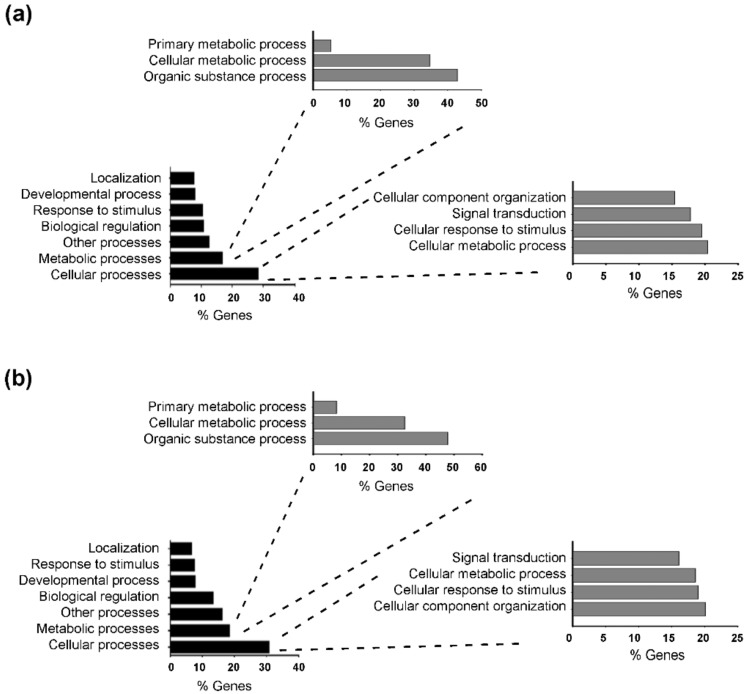
Starvation and T3 globally affect similar biological processes. (**a**) Gene ontology classification of the biological processes most affected by starvation in skeletal muscles. (**b**) Gene ontology classification of the biological processes most affected by T3 in STVT3 skeletal muscles.

To identify the molecular pathway modulated by T3 in starvation-induced muscle atrophy, we performed a transcriptome analysis of TA muscles, after 24 h of starvation. Total RNA was isolated from triplicates of CTR, STV, and STVT3 samples, and subjected to RNA sequencing analysis. By using 1.5 fold cut-off values for gene expression changes between CTR and STV, a total of 970 genes resulted significantly modulated by food deprivation (*p*-value < 0.05). As positive controls of the RNA-sequencing analyses, we checked and found genes known to be up-regulated by starvation in skeletal muscle, as atrogin-1, and MuRF-1, in the list of the up-regulated genes. Gene ontology analysis revealed that food deprivation mainly affected cellular processes (with 20.4% of genes involved in cell cellular metabolic process, followed by 19.5% of genes involved in cellular responses to stimulus) and metabolic processes (mainly organic substance metabolic process and cellular metabolic process), compared to controls (Figure 5a). When comparing STV with STVT3 samples, the thyroid hormone significantly modulated 664 genes (*p*-value < 0.05), by using 1.5-fold cut-off values for gene expression changes. Alike the previous data, gene ontology analysis revealed that T3 mostly affected cellular processes (with 20.1% of genes involved in cellular component organization, 19.0% in cellular response to stimulus, and 18.6% of genes involved in cellular metabolic process) and metabolic processes (mainly organic substance metabolic process and cellular metabolic process) in starved muscles (Figure 5b).

### 2.5. Thyroid Hormone Induces Metabolic Adaptation in Skeletal Muscle

**Figure 6:**
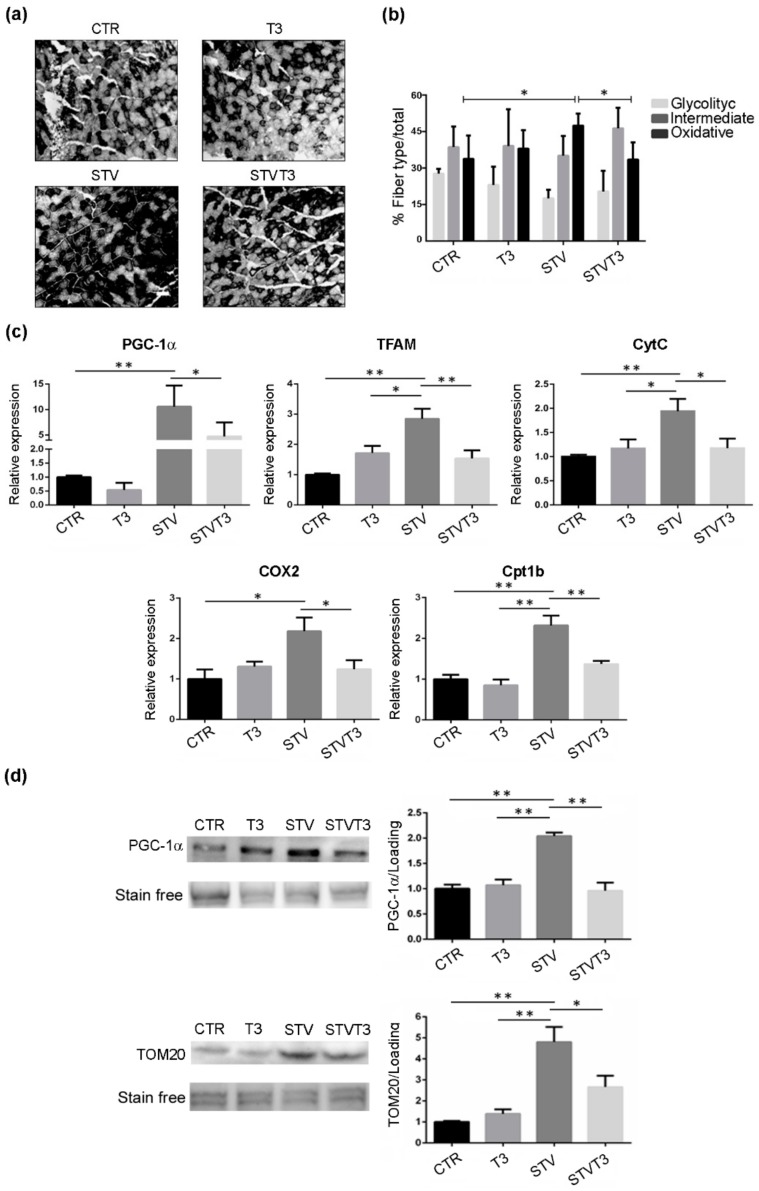
T3 modulates skeletal muscle metabolism following starvation. (**a**) Representative pictures of TA muscles stained with NADH, 48 h after treatments. Scale bar = 20 μm. (**b**) Quantification of oxidative, intermediate, and glycolytic myofibers, relative to total fibers, 48 h after treatments. *n* = 4 mice per each condition. Data are presented as means ± SD. Only for oxidative fibers: STV and T3 significantly interact (*p* = 0.009) by two-way ANOVA; * *p* < 0.05 with post hoc Tukey’s HSD test. (**c**) Expression levels of indicated genes by real-time PCR, 24 h after treatments. *n* = 6 mice per each condition. Data are presented as means ± SD. For PGC-1α: STV and T3 significantly interact (*p* = 0.039) by two-way ANOVA, * *p* < 0.05 and ** *p* < 0.01 with post hoc Tukey’s HSD test; for TFAM: STV has a significant effect (*p* < 0.0001), STV and T3 significantly interact (*p* < 0.0001), * *p* < 0.05 and ** *p* < 0.01 with post hoc Tukey’s HSD test; for CytC: STV has a significant effect (*p* = 0.019), STV and T3 significantly interact (*p* = 0.012), * *p* < 0.05 and ** *p* < 0.01 with post hoc Tukey’s HSD test; for COX2: STV has a significant effect (*p* = 0.026), STV and T3 significantly interact (*p* = 0.008), * *p* < 0.05 with post hoc Tukey’s HSD test; for Cpt1b: STV has a significant effect (*p* < 0.0001), STV and T3 significantly interact (*p* = 0.038), ** *p* < 0.01 with post hoc Tukey’s HSD test. (**d**) Representative blots and relative densitometric analyses of PGC-1α and TOM20 protein levels in TA, 24 h after treatments. Stain-free protein bands were used as a loading control. *n* = 4 mice per each condition. Data are presented as means ± SEM. For PGC-1α:STV has a significant effect (*p* = 0.0012), STV and T3 significantly interact (*p* = 0.0002), ** *p* < 0.01 with post hoc Tukey’s HSD test; for TOM20: STV has a significant effect (*p* < 0.0001), STV and T3 significantly interact (*p* = 0.0064) by two-way ANOVA, * *p* < 0.05 and ** *p* < 0.01 with post hoc Tukey’s HSD test.

To analyze the possible metabolic changes induced by T3 in our model, we performed NADH staining of TA histological sections and morphometric evaluation of the number of glycolytic, intermediate, or oxidative fibers. Histological analysis revealed important differences among samples (Figure 6a). Morphometric analyses showed that no significant changes in the number of glycolytic or intermediate fibers were detected among treatments. As for the number of oxidative fibers, instead, a significant increase was quantified in STV mice, respect to CTR. Interestingly, T3 significantly reduced the number of oxidative myofibers induced by STV (Figure 6b). 

The shift in fiber metabolism was further confirmed by the expression levels of several mitochondrial markers. Indeed, the expression of PGC-1α, a marker of mitochondriogenesis whose content correlates with the oxidative status of skeletal muscle [48], TFAM, CytC, and Cox2 were significantly increased by STV, with respect to CTR muscles, while T3 prevented the up-regulation triggered by STV (Figure 6c). Coherently, STV significantly increased also carnitine palmitoyltransferase 1B (Cpt1b) expression, a mitochondrial enzyme crucial for the beta-oxidation of long-chain fatty acids [49], while T3 prevented the STV-induced up-regulation (Figure 6c). Importantly, T3 per se did not significantly affect the expression of any of these genes (Figure 6c). 

These findings were corroborated with western blot analyses for PGC-1α and TOM20, which confirmed that T3 prevented the starvation-dependent increase in mitochondrial markers (Figure 6d and Figure S2e).

Muscle metabolism may correlate with Myh expression. Therefore, we quantified the expression of Myh7, Myh2, Myh1, and Myh4, by real-time PCR in our conditions. The metabolic reprogramming induced by starvation or T3 did not correlate with a concomitant change in Myh expression (Figure S3). Therefore, the T3-mediated resistance to STV-induced muscle atrophy depends on a different response of skeletal muscle metabolism.

## 3. Discussion

Muscle wasting is a devastating complication of several myopathies and systemic disorders, including chronic obstructive pulmonary disease, cystic fibrosis, cancer, or diabetes, and may be present in aging [18]. In pathological conditions, or during fasting, skeletal muscle is a valid source of metabolites and amino acids that can be used for energy production by the skeletal muscle itself, as well as by other organs, including heart, liver, and brain. Skeletal muscle atrophy is characterized by the reduction in muscle mass, fiber cross-sectional area, protein content, and strength, leading to increased fatigability and insulin resistance [50]. Importantly, loss of muscle mass directly correlates with poor prognosis in several diseases and can impair the efficacy of treatments. Thus, muscle atrophy contributes to morbidity and mortality in many pathological conditions, highlighting the importance of targeting skeletal muscle with pharmacological approaches. 

Anorexia, or loss of appetite, is a common concomitant factor in many diseases, including cancer or AIDS [51,52,53]. Starvation-induced skeletal muscle atrophy, which mimics anorexia, is characterized by an increase in the rates of protein catabolism with a reduction in muscle protein synthesis [53]. Searching for treatments aiming to counteract starvation-induced muscle atrophy, we investigated T3, an important regulator of skeletal muscle metabolism and homeostasis [38]. Hyperthyroidism increases metabolism, oxygen consumption, and heat generation; conversely, hypothyroidism has been associated with a decrease in metabolic rate [54]. Importantly, in our experimental conditions, administration of T3 significantly affected the expression of numerous well-known T3-target genes in skeletal muscle, without increasing the Dio2 expression levels in skeletal muscle, which resulted significantly down-regulated by starvation, as previously reported [55], or the free T3 levels in sera, as expected considering the short T3 half-life (about 12–18 hours) [56,57].

While STV significantly reduced the mass, the whole muscle, and the myofiber CSA of two distinct muscles, such as TA and soleus, T3-treatment preserves muscle mass and CSA despite the pro-atrophic stimulus. The major contribution to protein degradation in muscle usually depends on the activation of the ubiquitin-proteasome pathway [58]. In addition to the proteasome, autophagy is increased upon starvation or caloric restriction, contributing to the development of muscle atrophy [59,60]. FOXO3 is an upstream regulator of both proteasomal and autophagic degradation pathways in skeletal muscle [61]. It is interesting to note that, while T3 treatment prevented starvation-induced muscle atrophy, it did not interfere with FOXO3, proteasome, or autophagic activation following the pro-atrophic stimulus. Being markers of muscle atrophy, but also required for skeletal muscle atrophy [26,62], the expression of atrogin-1 and MuRF1 was quantified in our experimental conditions. Consistently with the proteasome activity data, the expression levels of the two muscle E3 ubiquitin-ligases were not significantly modulated by T3 following starvation. Similarly, the expression and protein levels of the two autophagic markers LC3b and p62 were not altered by T3 in skeletal muscle of starved mice, confirming that the T3-mediated effects on muscle mass were not dependent on impairment in the activation of the catabolic pathways. 

Treatments aimed to counteract muscle wasting generally enhance muscle regeneration [63,64]. Thyroid hormone has been demonstrated to regulate myogenesis and muscle regeneration, as well as the maintenance of the satellite cell pool in muscle wasting and aging [44,65,66]. However, high levels of T3 inhibit satellite cell proliferation, promoting premature differentiation, and hampering muscle regeneration [67]. Moreover, the thyroid hormone has been demonstrated to induce the phosphoinositide 3-kinase/Akt signaling in other tissues [5,6,7,8], which may contribute to preventing starvation-induced muscle atrophy. Therefore, we analyzed muscle anabolism, Akt activation, and de novo myogenesis markers in our experimental conditions, finding that T3 treatment did not increase myogenesis, protein synthesis, or satellite cell activation in starved or control skeletal muscle. Probably thyroid hormone can potentiate muscle regeneration once satellite cells are activated, but T3 administration does not trigger muscle regeneration per se or following starvation in skeletal muscle.

Aiming to understand the molecular mechanisms underlying the T3-mediated resistance to starvation-induced skeletal muscle atrophy, we performed a transcriptome analysis by comparing control, starved and T3-treated starved muscles, since the majority of cellular alterations after T3 treatment are mediated by the transcriptional regulation of gene expression through thyroid hormone receptors, rather than by non-genomic actions [68]. Interestingly, “cellular and metabolic processes” were the biological processes most affected by both starvation and T3 in skeletal muscle. While a modulation in metabolic processes was expected as a result of starvation [21,69], it was intriguing that the same biological process was altered when comparing STV to STVT3 muscles. We then analyzed muscle metabolism by NADH staining, a measure of the myofiber oxidative capacity. Starvation induced a metabolic shift, characterized by a significant induction in the number the slow-oxidative fibers, as previously reported [70,71]. This data was also corroborated by the induction of the PGC-1α and TOM20 gene and protein expression, as well as by the expression of other mitochondrial markers, i.e., TFAM, CytC, COX2, and Cpt1b, which are involved in the mitochondrial biogenesis, oxidative phosphorylation, and fatty acid oxidation and reflect the STV-mediated structural/metabolic shift in skeletal muscle [72,73]. Strikingly, T3 treatment prevented the STV-mediated increase in the number of oxidative fibers and the induction of the expression of all these markers, preserving a fiber type composition similar to that of control fed mice. Accordingly, the thyroid hormone is known to induce a shift to faster contractile function, by promoting the expression of fast genes [74] and by repressing the expression and the activity of calcineurin, an important regulator of the slow muscle phenotype [13]. Interestingly, the metabolic shift induced by either starvation or thyroid hormone was not accompanied by changes in fiber type-specific myosin heavy chain isoform expression, a phenomenon already described in another context [75]. These data indicate that the metabolic shift mediates starvation-induced muscle atrophy and that T3 counteracts muscle atrophy by modulating muscle metabolism. 

Numerous studies explored the mechanisms involved in the regulation of skeletal muscle mass, with the long-term goal to improve the prognosis of many diseases or the quality of life in aging through hindering muscle atrophy. Several potential pharmacological targets have been identified, though no effective treatment to counteract muscle wasting is yet available. Proteasome inhibitors efficiently rescued skeletal muscle atrophy in mice [76,77], although prolonged inhibition is detrimental for skeletal muscle [78]. Similarly, a proper autophagic response is crucial for the maintenance of skeletal muscle homeostasis, both in physiological and in pathological conditions [29,78,79,80]. Skeletal muscle metabolism has been identified as a potential target in different models of muscle atrophy or sarcopenia, with pharmacological treatments aimed to induce a metabolic shift mimicking that of exercise [81,82,83,84]. Our findings are significant in the light of possible clinical implications, considering that several pathological conditions are characterized by a metabolic shift, such as sepsis, cachexia, denervation, acute diabetes, or glucocorticoids [85]. In these contexts, counteracting the metabolic shift may be beneficial for maintaining muscle mass. 

## 4. Materials and Methods

### 4.1. Animals and Sample Collection

Adult (10-week-old) male Balb/c mice were used. Mice were treated in strict accordance with the guidelines of the Institutional Animal Care and Use Committee and to national and European legislation, throughout the experiments. All animal experiments were performed according to animal care and welfare animal protocol approved by the Italian Ministry of Health (authorization # 138/2016-PR). Mice were housed in cages in an environmentally controlled room (23 °C, 12-h light-dark cycle) and provided food and water ad libitum. Fasting was chosen as a method of induction of atrophy condition: after being weighed, first in the morning, the animals were food-deprived for 24 or 48 h and daily injected intraperitoneally with (100 μg/kg BW) T3 (stock solution 10^−3^ M in NaOH) (Sigma Aldrich, St Louis, MO, USA) or vehicle (0.95% NaCl) as controls, as previously done [39].

Mice were sacrificed by cervical dislocation after 24 or 48 h to perform molecular biology or histological and morphometric analyses. Blood was collected after 48 hours of treatments, plasma was separated by centrifugation, and stored at −20 °C. 

### 4.2. Histology 

TA and soleus muscles were dissected, embedded in tissue freezing medium (Leica, Wetzlar, Germany) and frozen in liquid nitrogen-cooled isopentane. Cryosections (5–7 µm) were obtained from the mid-belly of the muscles using a Leica cryostat (Leica Biosystems, Nussloch GmbH 2019, Germany). 

#### 4.2.1. Histological Sample Preparation

Cryosections were stained with hematoxylin and eosin (Sigma Aldrich, St Louis, MO, USA) using a standard procedure. NADH-transferase staining was used to distinguish different fiber type metabolism and was performed by using standard methods. 

#### 4.2.2. Immunofluorescence

For Pax7 and Laminin, cryosections were fixed in 4% paraformaldehyde buffered solution for 10 min at room temperature and permeabilized with absolute methanol for 6 min at −20 °C. Then, cryosections were heat-activated with 0.01  M of citrate buffer pH 6.0 for 10  min. Samples were then blocked for three hours with 4% BSA in PBS and incubated overnight with 1:20 anti-Pax7 antibody (mouse monoclonal, Developmental Studies Hybridoma Bank) and with 1:100 rabbit polyclonal anti-Laminin antibody (Sigma Aldrich, L9393). After washing in 0.1% BSA/PBS, cryosections were incubated with 1:1000 biotin-conjugated secondary antibody (Jackson ImmunoResearch, Pennsylvania, USA) and with 1:500 dilution of anti-rabbit-Alexa 488 (Life Technologies, Carlsbad, CA, United States) secondary antibodies for 45 min at room temperature. Then the samples were incubated with 1:2500 streptavidin antibody (Jackson ImmunoResearch) for 30 min at room temperature. Nuclei were counterstained with Hoechst 0.5 μg/mL, and samples were mounted with 60% glycerol in Tris HCl 0.2 M pH 9.3.

For Laminin, cryosections were fixed in 4% paraformaldehyde for 10 min at room temperature, then washed and blocked with 1% BSA (Sigma, St. Louis, MO, USA) for 30 min. Samples were then incubated with a 1:100 rabbit polyclonal anti-laminin antibody (Sigma Aldrich, L9393) in 1% BSA overnight at 4 °C. To detect the primary antibody, we incubated the samples with a 1:500 dilution of anti-rabbit-Alexa 488 (Life Technologies) secondary antibodies in 1% BSA for 1 h at room temperature.

### 4.3. Morphometric Analysis

Photographs were acquired using an Axio imager A2 system equipped with an Axiocam HRc, with Axiovision Release 4.8.2 software (Zeiss, Oberkochem, Germany), at standard 1300 × 1030 pixel resolution. The whole-muscle CSA or the myofiber CSA of the entire cryosection was quantified from the mid-belly of TA and soleus muscles on hematoxylin and eosin sections by using ImageJ software. 

The amounts of type IIb (low NADH transferase activity, glycolytic), type IIa/x (medium NADH transferase activity, intermediate), and type I (high NADH transferase activity, oxidative) TA fibers of the entire cryosection were counted. For each muscle, the fiber type number was normalized for the total number of the fibers, to calculate the percentage of fiber types. 

The whole cross-section of each muscle was analyzed to quantify the myofiber CSA or the % of fiber type (between 900 and 1300 fibers per TA muscle and between 600 and 900 fibers per soleus).

### 4.4. RNA Isolation and Quantitative RT-PCR Analysis

Total RNA was extracted from muscle tissue samples by TRIzol (Invitrogen, Carlsbad, CA, USA) following the manufacturer’s protocol. One μg of RNA was retrotranscribed using the High-Capacity cDNA kit reverse transcription kit (Applied Biosystem, Foster City, CA, USA). Real-time PCR was performed by using the Step One Plus Real-time PCR System (Applied) tool and Power- SYBR^®^ Green PCR Master Mix (Applied Biosystems) following the manufacturer’s instructions. 18S was used to normalize gene expression. Primers are listed in Table 2 and were synthesized by BioFab Research Srl.

### 4.5. RNA Sequencing 

Total RNA was extracted from TA muscles by using Trizol and purified by using the RNeasy MinElute Cleanup Kit (Qiagen, Hilden, Germany). For each sample, 2 µg of RNA were shipped to the IGA Technology Services company (Udine, Italy). “TruSeq Stranded mRNA Sample Prep” (Illumina, San Diego, CA, USA) was used to prepare the library following the manufacturer’s instructions. Libraries were processed with Illumina cBot for cluster generation on the flow cell and sequenced on single-end mode at the multiplexing level requested on HiSeq. 2500 (Illumina, San Diego, CA, USA). DNA libraries of different samples pooled in the same sequencing tube were uniquely distinguished by the addition of a specific barcode sequence; by identifying the barcode sequence, it was then possible to proceed to de-multiplexing of the read and aligning them with reference sequences in the databases to obtain the real sequence of each analyzed sample. All the resulting alignments were provided to Cufflinks [86] to generate a transcript of the transcriptome for each experimental condition. These transcripts were then fused using the Cuffmerge included in the Cufflinks package and provide the basis for calculating gene expression in any condition. The read and the transcripts were also supplied to the Cuffdiff software that calculates the gene expression levels and tests the statistical significance of the observed variations. 

### 4.6. Bioinformatics Analysis

RNA-Seq standard bioinformatics analysis was performed by the IGA Technology Services company, which included base calling and de-multiplexing, Trimming (removing lower quality bases and adapters) using ERNE [87] and Cutadapt [88] software, alignments with TopHat2 [86,89], pair-wise differential expression analysis (Cuffdiff) [90]. From the Cuffdiff gene list, those genes that were significantly modulated between the different treatment conditions (*p* < 0.05) were extrapolated. These genes, each labeled with an ID, were subsequently subjected to bioinformatics analysis using an available on-line browser (http://www.pantherdb.org/) (Protein ANalysis THrough Evolutionary Relationships), a biological database of gene and protein families that identifies and classifies the functions of gene products. 

### 4.7. Proteasome Assay

TA muscles were isolated, minced and homogenized in Lysis Buffer (50 Mm Tris HCl pH 7.4, 1 mM EDTA, 150 Mm NaCl, 1% Triton, supplemented with protease and phosphatase inhibitors). 10 μg of freshly isolated protein extracts were used for measuring proteasome activity, by using the Chemicon Proteasome Activity Assay Kit (APT280), following manufacturer’s instructions. Cleavage activity was quantified by using a fluorescence plate reader (Infinite F200 PRO, TECAN, Männedorf, Switzerland) at 360/460 nm.

### 4.8. Protein Extraction and Western Blot

Muscles were dissected, minced, and homogenized in lysis buffer (50 Mm Tris HCl pH 7.4, 1 mM EDTA, 150 Mm NaCl, 1% Triton) supplemented with protease and phosphatase inhibitors. Protein concentration was quantified using the BCA method (BCA Protein Assay Kit, Thermo Fisher Scientific). For each sample, 40–70 μg of proteins were run on the nUView Tris-Glycine gels. Gels were transferred to nitrocellulose membranes, and protein bands were visualized under UV light, without staining. The whole signal of the entire lane has been used for loading and transfer control. Membranes were blocked with 5% milk or 5% BSA in TBST and blotted with different primary antibodies. After washing in TBST, membranes were incubated with HRP secondary antibodies conjugates (BIO-RAD 170-6515 or 170-6516), and signals were detected by using ECL chemistry (Cyanagen XLS3,0100). Images were acquired on a ChemiDoc MP imaging system (BIO-RAD, Hercules, CA, USA) with Image Lab 5.2.1 software. The following primary antibodies were used: puromycin (Sigma-Aldrich clone 12D10 #MABE343), pAKT Ser 473 (Cell Signaling #9271), Akt (Cell Signaling #9272), pFoxo3a Ser 253 (Cell Signaling #9466), Foxo3a (Cell Signaling #2497), LC3b (Cell Signaling #2775), p62 (Sigma-Aldrich #P0067), TOM20 (Santacruz #sc11415), and PGC1-α (Abcam #ab54481).

### 4.9. SUnSET Assay

After 48 h of starvation, mice were injected with puromycin (0.04 µmol/g BW) and sacrificed 30 min later. TA muscles were isolated, and proteins were extracted as described above. Protein synthesis was evaluated by Western blot with an anti-puromycin antibody (Sigma-Aldrich clone 12D10 #MABE343), as described in [46].

### 4.10. ELISA

Free T3 present in the plasma was quantified using a competitive immunoassay ELISAs with colorimetric detection (Abnova Corporation # KA0199), following the manufacturer’s instructions.

### 4.11. Statistics

Data are expressed as mean +/− standard deviation (SD) or +/− SEM. Statistical significance was determined by using either Student’s t-test or two-way analysis of variance (ANOVA) followed by post hoc Tukey’s HSD test, if one or two variables were compared, respectively. VassarStats (http://vassarstats.net/) was used for calculating ANOVA.

## 5. Conclusions

In conclusion, the T3 thyroid hormone affects skeletal muscle metabolism, counteracting starvation-induced muscle atrophy. Our results offer new and exciting perspectives to the field, introducing a potential approach for the pharmacological treatment of skeletal muscle atrophy and setting the bases for future applications based on thyroid hormone administration.

## Figures and Tables

**Table 1:** Validation of T3 administration. Expression levels of T3-target genes in skeletal muscle of controls and T3-treated mice. Data are expressed as mean ± standard deviation. *n* = 4 per each condition. * *p* < 0.05; ** *p* < 0.005 by Student’s *t*-test.

Gene	CTR	T3
*GLUT 4*	1 ± 0.15	1.83 ± 0.45 *
*UCP3*	1 ± 0.33	2.14 ± 0.23 *
*MyoD*	1 ± 0.32	1.5 ± 0.22 *
*Myh7*	1 ± 0.20	0.60 ± 0.15 *
*Myh1*	1 ± 0.29	3.40 ± 0.61 **

**Table 2:** Primers used for real-time PCR.

Gene	Sequence	
*Atrogin-1*	**F:** GCA AAC ACT GCC ACA TTC TCT C	
	**R:** CCT GAG GGG AAG TGA GAC G	
*MuRF-1*	**F:** ACC TGC TGG TGG AAA ACA TC	
	**R:** CTT CGT GTT CCT TGC ACA TC	
*LC3b*	**F:** CAC TGC TCT GTC TTG TGT AGG TTG	
	**R:** TCG TTG TGC CTT TAT TAG TGC ATC	
*P62*	**F:** CCC AGT GTC TTG GCA TTC TT	
	**R:** AGG GAA AGC AGA GGA AGC TC	
*Pax7*	**F:** TCC CCC TGG AAG TGT CCA	
	**R:** TGG GAA ACA CGG AGC TGA	
*Myh3*	**F:** AGC AGC TCA ATC AGC TCA	
	**R:** TGG TCG TAA TCA GCA GCA	
*PGC-1α*	**F:** TGA GTA ACC GGA GGC ATT CTC T	
	**R:** TGA GGA CCG CTA GCA AGT TTG	
*Dio2*	**F:** CCT CCT AGA TGC CTA CAA ACAG	
	**R:** TGA TTC AGG ATT GGA GAC GTG	
*Myh7*	**F:** AGT CCC AGG TCA ACA AGC TG	
	**R:** TTC CAC CTA AAG GGC TGT TG	
*Myh2*	**F:** AGT CCC AGG TCA ACA AGC TG	
	**R:** GCA TGA CCA AAG GTT TCA CA	
*Myh1*	**F:** AGT CCC AGG TCA ACA AGC TG	
	**R:** CAC ATT TTG CTC ATC TCT TTG G	
*Myh4*	**F:** AGT CCC AGG TCA ACA AGC TG	
	**R:** TTT CTC CTG TCA CCT CTC AAC A	
*TFAM*	**F:** AAA GCT TCC AGG AGG CAA	
	**R:** GCC ATC TGC TCT TCC CAA	
*CytC*	**F:** CCA GTG CCA CAC TGT GGA	
	**R:** GTC TTC CGC CCG AAC AGA	
*COX2*	**F:** AAG ACG CCA CAT CCC CTA	
	**R:** CGT AGA GAG GGG AGA GCA	
*Cpt1b*	**F:** TCT CCA TGC GAC TGG TCG AT	
	**R:** GAG ACG GAC ACA GAT AGC CC	
*MyoD*	**F:** ACC CAG GAA CTG GGA ATG GA	
	**R:** AAG TCG TCT GCT GTC TCA AA	
*Glut4*	**F:** ATG GCT GTC GCT GGT TTC TC	
	**R:** ACC CAT ACG ATC CGC AAC AT	
*UCP3*	**F:** TGA CCT GCG CCC AGC	
	**R:** CCC AGG CGT ATC ATG GCT	
*18s*	**F:** GCA ATT ATT CCC CAT GAA CG	
	**R:** GGG ACT TAA TCA ACG CAA GC	

## Supplementary Materials

Supplementary materials can be found at https://www.mdpi.com/1422-0067/20/22/5754/s1.

## Abbreviations

THs	Thyroid hormones
TRs	Thyroid receptors
PGC-1α	Peroxisome proliferative activated receptor Gamma Coactivator 1 alpha
TA	Tibialis Anterior
ANOVA	Analysis of variance
STV	Starved
CTR	Control
Dio2	Type-2 iodothyronine deiodinase
CSA	Cross-sectional area
Cpt1b	Carnitine palmitoyltransferase 1B
