# Supplementary material for: Thyroid Hormone Protects from Fasting-Induced Skeletal Muscle Atrophy by Promoting Metabolic Adaptation
Source: Int J Mol Sci. 2019 Nov 15;20(22):5754. doi: 10.3390/ijms20225754 (PMC6888244; doi:10.3390/ijms20225754)
Supplement: Supplementary file 1 [file ijms-20-05754-s001.pdf]

Supplementary Figures

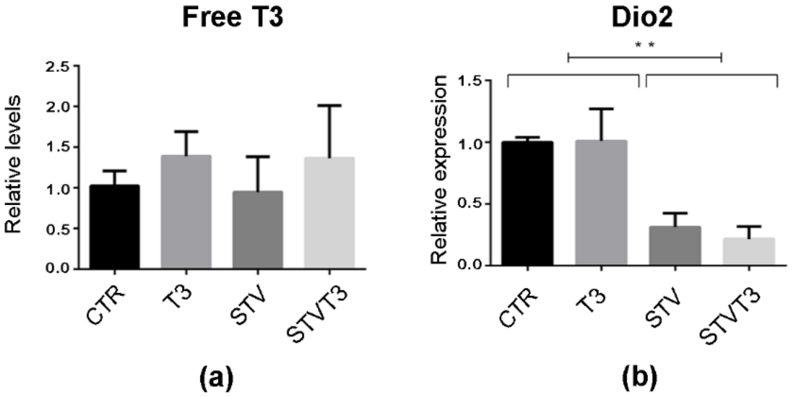

Figure S1: Thyroid hormone treatment does not affect circulating T3 levels or Dio2 expression in skeletal muscle. (**a**) Quantification of free T3 serum levels by ELISA assay, 48 hours after treatments. n=4 mice per each condition. Data are presented as means ± SD. (**b**) Dio2 mRNA expression by real time PCR, 24 hours after treatments. n=4 mice per each condition. Data are presented as means ± SD; STV has a significant effect: ** $p < 0.0001$ by two-way ANOVA.

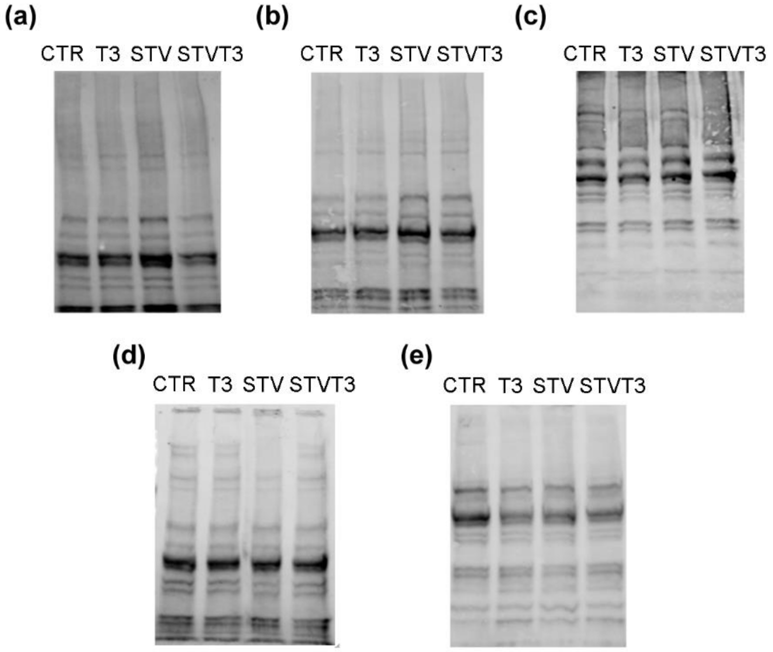

Figure S2: Stain-free blot membranes. (**a**) pFOXO3a and pAKT membrane; (**b**) FOXO3a and AKT membrane; (**c**) LC3 and p62 membrane; (**d**) Puromycin membrane; (**e**) TOM20 and PGC-1α membrane.

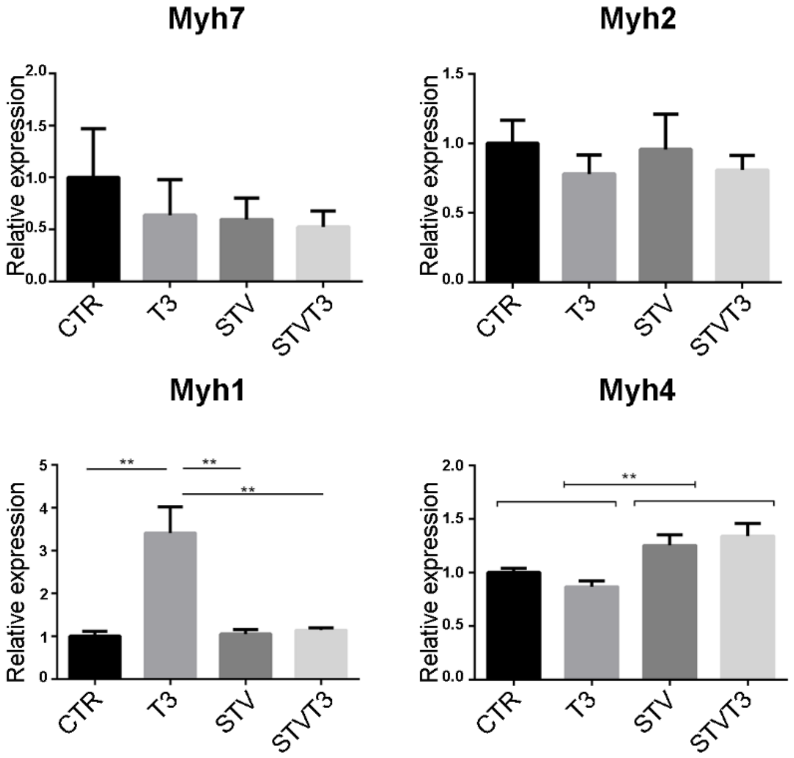

Figure S3: Metabolic shift does not correlate with changes in myosin heavy chain expression. Expression levels of indicated myosin heavy chain genes in TA, 24 hours after treatments. n=6 mice per each condition. Data are presented as means ± SD. For Myh1: T3 has a significant effect ($p<0.0001$); STV has a significant effect ($p = 0.0001$); and the two variables significantly interact ($p = 0.0001$) by two-way ANOVA; ** $p < 0.01$ by post-hoc Tukey's HSD test. For Myh4: STV has a significant effect ($p = 0.0007$) by two-way ANOVA.
